# Multimetal Research in Powder Bed Fusion: A Review

**DOI:** 10.3390/ma16124287

**Published:** 2023-06-09

**Authors:** Liming Yao, Aditya Ramesh, Zhongmin Xiao, Yang Chen, Quihui Zhuang

**Affiliations:** 1 State Key Laboratory of Robotics and Systems (HIT), Harbin 150000, China; 2 School of Mechanical and Aerospace Engineering, Nanyang Technological University, 50 Nanyang Avenue, Singapore 639798, Singapore; 3 School of Mechanical Engineering, Chongqing University of Technology, Chongqing 400054, China

**Keywords:** multimetal, additive manufacturing, dissimilar alloys, powder bed fusion, microstructure, melt pool flow

## Abstract

This article discusses the different forms of powder bed fusion (PBF) techniques, namely laser powder bed fusion (LPBF), electron beam powder bed fusion (EB-PBF) and large-area pulsed laser powder bed fusion (L-APBF). The challenges faced in multimetal additive manufacturing, including material compatibility, porosity, cracks, loss of alloying elements and oxide inclusions, have been extensively discussed. Solutions proposed to overcome these challenges include the optimization of printing parameters, the use of support structures, and post-processing techniques. Future research on metal composites, functionally graded materials, multi-alloy structures and materials with tailored properties are needed to address these challenges and improve the quality and reliability of the final product. The advancement of multimetal additive manufacturing can offer significant benefits for various industries.

## 1. Introduction

Powder bed fusion (PBF) is one of the advanced technologies for rapid and high-precision forming of metal parts that uses a laser or electron beam to selectively melt microscopic metal or alloy powder on a flat powder bed. This process is repeated layer by layer to fabricate three-dimensional (3D) parts [1,2]. Additionally, PBF can be employed for joining or cladding two dissimilar metals or alloys. However, when printing one metal or alloy layer onto a different metal or alloy base via PBF, the mismatch in thermophysical and chemical properties can result in iso-surface cracks [3,4] or detachment [5].

In contrast to their homogeneous counterparts, multimetal alloys exhibit remarkable mechanical characteristics, including enhanced fracture and fatigue resistance, remarkable strain hardening, strength–ductility synergy, and exceptional resistance to wear and corrosion [6,7]. Consequently, to address the thermo-mechanical and strength–ductility trade-off in metallic materials, gradient nanostructured (GNS) metals were introduced in the mid-2000s. The presence of a structural gradient in these materials led to an improvement in various mechanical properties such as high strength, significant ductility, a high rate of work hardening, improved fatigue resistance, and enhanced friction properties [8,9]. Multimetal or functionally graded materials (FGM) find extensive applications in numerous industries, including aerospace, automotive, nuclear, marine, and biomedical, particularly for lightweight components. Powder bed fusion technology serves as an effective and efficient method for the rapid and high-precision manufacturing of multimetal parts.

**Figure 1:**
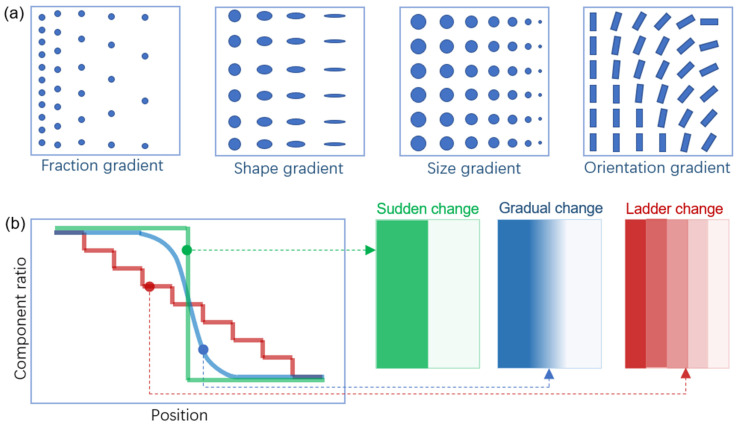
(**a**) Different types of gradients in functionally graded material (FGM); (**b**) showing schematic illustration of sudden-change, gradual-change, and ladder-change FGMs, respectively [10].

Multimetal/FGM is distinguished by progressive changes in either composition/constituents or microstructure (for example, grain size, texture, porosity, etc.) in at least one direction, thereby causing functional changes connected to at least one attribute. As illustrated in Figure 1, FGM is divided into four categories according to the nature of their gradients: fraction gradient, orientation gradient, shape gradient, and size gradient type. Furthermore, if the composition and/or microstructure change occurs in a stepwise manner, functionally graded materials can be distinguished as sudden-change, gradual-change, or ladder-change FGMs [10].

Multimetal PBF is a relatively new field of research and many researchers are exploring the potential applications of this technique. PBF techniques such as selective laser melting (SLM) and electron beam melting (EBM) can be used to fabricate multimetallic components [11]. The mechanical properties, material characteristics, and microstructure of multimetal/FGM can all be controlled by selecting the proper fabrication technique and constituent materials [12]. The current state of research in multimetal PBF can be broadly divided into three areas:

(a) Material development. The development of new materials that can be used in multimetal PBF is an active area of research. Many researchers are exploring the potential of new alloys and composites that can be used in this technique. The goal is to develop materials with improved properties such as corrosion resistance, wear resistance, and biocompatibility.
(b) Process development. The development of new processes and process parameters is crucial for the success of multimetal PBF. Many researchers are exploring the potential of new printing methods. Furthermore, researchers are investigating the effect of process parameters such as laser power, scan speed, and powder bed temperature on the final product.
(c) Application development. The application of multimetal PBF is not limited to a particular field. Many researchers are exploring the potential of this technique in various fields such as aerospace, automotive, and biomedical engineering. The aim is to find new applications that can benefit from the unique advantages of multimetal PBF.

Multimetal PBF is a relatively new and rapidly advancing technology in the field of additive manufacturing. As with any developing field, there have been significant advancements, discoveries, and innovations in multimetal PBF over the last few years. This review paper on multimetal PBF provides an updated, comprehensive, and consolidated resource that reflects the latest advancements, research findings, practical applications, and comparative analyses in this rapidly evolving field of additive manufacturing. In the following sections, the research progress, challenges, and future prospects of multimetal PBF will be introduced in detail.

## 2. Multimetal Additive Manufacturing Techniques

Powder bed fusion (PBF) technology is a mainstream manufacturing technology for multimetal 3D printing. PBF can be further subdivided into laser powder bed fusion (L-PBF), electron beam powder bed fusion (EB-PBF), and large-area pulsed laser powder bed fusion (L-APBF) based on its energy sources [13].

### 2.1. Laser Powder Bed Fusion (LPBF)

Laser powder bed fusion (LPBF) is an additive manufacturing (AM) process that uses laser as a thermal energy source to melt materials. A blade or wiper mechanism applies a layer of powder material to the build plate in accordance with the cross-sectional slice of the 3D CAD model. A laser beam of high intensity heats the layer, and the melt pool is formed, and subsequently solidified by rapid cooling. After the laser exposure, the build platform is lowered by a depth equal to the desired layer thickness. The layer thickness in LPBF is a crucial parameter that affects the overall resolution, accuracy, and surface finish of the final printed part. Thinner layers result in finer detail and smoother surfaces but may increase processing time due to the higher number of layers required. Thicker layers can reduce processing time but may compromise surface quality and part accuracy. Lowering the build platform by the specified layer thickness allows the next layer of metal powder to be uniformly distributed on top of the previous layer [14]. This procedure is repeated in an inert environment with a fresh layer of powder until the desired 3D CAD model is obtained. The laser intensity, scan rate, hatch spacing, and layer thickness are some of the parameters associated with the LPBF process [15]. A few different forms of L-PBF are direct metal laser sintering (DMLS), selective laser sintering (SLS), and selection laser melting (SLM).

**Figure 2:**
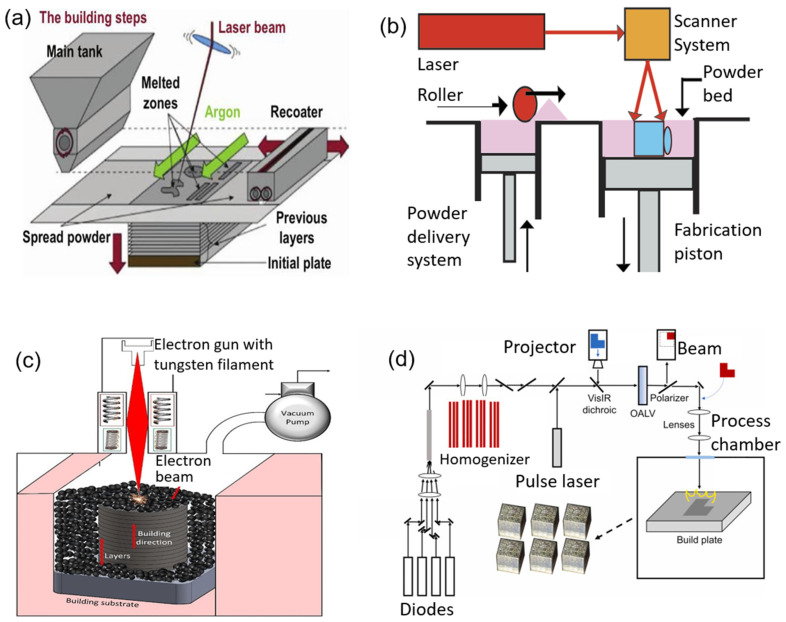
Schematics of (**a**) SLM, (**b**) SLS, (**c**) EB-PBF and (**d**) L-APBF processes.

SLM is a process that occurs in a highly pressurized chamber and inert environment to reduce the amount of oxygen during the process. A wide range of materials can be produced additively using SLM, and the mechanical properties of the component can be modified by adjusting their process parameters. The impact of different material qualities on the printability of the multimetal 3D-printing processes were examined by Hasanov et al. and Guan et al. [16,17]. On the basis of a predetermined process setting and scanning tactics, Chen et al. explored the fabrication of 316L/CuSn10 bimetallic structures printed via SLM. Chueh et al. printed copper alloy powder (Cu10Sn) and nylon powder (PA11) using a unique multimetal LPBF machine. A layer of polymer powder is applied to the build platform using an ultrasonic vibration-assisted nozzle (UVAN), and any leftover powder is cleared away using a powder-removing nozzle [18,19]. Advantages of SLM include using a wide range of materials, an increased functionality, the production of net-shaped components, and being relatively inexpensive. Disadvantages of SLM include strict size restrictions, challenging powder handling, high power consumption, and the potential for rough surfaces on finished parts [20]. Figure 2a shows the schematics of an SLM system.

The SLS system has three compartments, the part bed being the central compartment. Powder stored in the other two compartments is transported over the build platform via a roller. Before sintering, the powder bed is preheated to just below the material’s melting point. Material and process parameters affect the structural and mechanical strength of SLS-produced components, with the energy density and laser scanning speed being particularly important. SLS is an economical and functional process for the rapid prototyping and development of new materials and products, with high part accuracy and versatility [21]. Figure 2b shows the schematics of an SLS system.

### 2.2. Electron Beam Powder Bed Fusion (EB-PBF)

Electron beam powder bed fusion (EB-PBF) is a 3D-printing technique that uses an electron beam as the energy source to fuse layers of powder. The process requires high temperatures and overnight cooling periods. The electron beam melting (EBM) process involves process variables such as beam power, line spacing, focus diameter, scanning velocity, plate temperature, and scan strategy. Using the Arcam A2 EBM system that can only process one material at a time, Terrazas et al. [22] created multimaterial Ti-6Al-4V and copper samples. The process parameters caused changes in the hardness and microstructure of copper and Ti-6Al-4V. According to recent studies, multimaterial metallic components made of stainless steel, copper, Inconel 718, and other materials can be produced using EBM technology. The EBM technique is suitable for producing brittle materials as it avoids solidification cracks by gradually cooling the melt. However, it is less common than SLM due to its higher equipment cost, poor precision, and lack of huge build-up quantities [23]. Figure 2c shows the schematics of an EBM system.

### 2.3. Large-Area Pulsed Laser Powder Bed Fusion (L-APBF)

Large-area pulsed laser powder bed fusion (L-APBF) is a new technique that uses laser diodes to warm the metal powder before a strong laser pulse melts it into the substrate. It also uses an optically addressed light valve (OALV) to pattern lasers with high resolution. L-APBF has the potential to provide high build rates without losing printed-part resolution, with current machines producing a build rate of 90 cm^3^/h. Benefits of L-APBF include lower residual stress and less material ejection compared to conventional LPBF. However, more research is needed to determine the benefits and drawbacks of the method [24]. Figure 2d shows the schematics of an L-APBF system.

## 3. Multimetal Research Based on Powder Bed Fusion

### 3.1. Experiment

Interface characterization involves studying the interaction and bonding between the successive layers in the printed part. In PBF, each layer is built upon the previous layer, and the quality of the interfaces between these layers is critical for achieving mechanical integrity and performance. The surface morphology of the interfaces can be analyzed using techniques such as optical microscopy and scanning electron microscopy (SEM). These techniques can reveal the presence of defects such as pores, cracks, or incomplete fusion that can weaken the bond between the layers. Microstructural analysis techniques, such as SEM or transmission electron microscopy (TEM), can provide information about the microstructure at the interface. This includes the grain structure, distribution of phases, and any changes in microstructure caused by the heat input during the printing process. Techniques such as energy-dispersive X-ray spectroscopy (EDS) or X-ray diffraction (XRD) can be used to analyse the elemental composition and identify any intermetallic compounds that may have formed at the interface. The presence of intermetallics can affect the mechanical properties and integrity of the printed part. Diffusion refers to the movement of atoms or molecules from regions of high concentration to regions of low concentration. In the context of PBF, diffusion occurs during the melting and solidification of the metal powder particles, and it affects the microstructure and properties of the printed part. The diffusion process in PBF is influenced by various factors. During the laser or electron beam scanning process, the localized heating and subsequent cooling creates a temperature gradient in the material. This temperature gradient drives the diffusion of atoms or solute species, which can lead to the redistribution of elements and formation of concentration gradients [25]. The melting and solidification process during PBF can induce diffusion at the interface between the liquid and solid phases. As the molten metal solidifies, solute atoms or impurities can segregate or diffuse towards the interface, thereby affecting the local microstructure and properties. The diffusion of alloying elements can also occur during the melting and mixing of different powders, leading to the formation of alloy gradients or interdiffusion zones. Characterizing diffusion in PBF involves techniques such as electron microscopy, atom probe tomography (APT), or secondary ion mass spectrometry (SIMS). These techniques can provide insights into the diffusion paths, diffusion rates, and composition profiles across interfaces. Understanding the mechanisms of interface characterization and diffusion in PBF is crucial for optimizing process parameters, minimizing defects, and tailoring the microstructure and properties of additively manufactured components. By analysing the interfaces and diffusion phenomena, researchers and engineers can enhance the quality, reliability, and performance of 3D-printed metallic parts [26,27].

In multimetal PBF, the formation of an unmixed zone at the interface refers to a distinct region where two different metals or alloys do not fully mix during the printing process. This occurs due to the differences in thermal and chemical properties between the metals being printed. The primary reason for the formation of the unmixed zone is the limited diffusion between the metals at the interface during the short time available for solidification. The diffusion distance is governed by the temperature gradient and the diffusion coefficients of the metals involved. If the metals have significantly different diffusion coefficients or if the temperature gradient is large, the diffusion may be insufficient for complete mixing at the interface. As a result, an unmixed zone which forms between the two metals is characterized by a distinct boundary where each metal maintains its composition. This unmixed zone can have different microstructural features and properties as compared to the bulk materials. It may contain intermetallic compounds, residual stresses, or compositional gradients. The formation of the unmixed zone can have both advantages and disadvantages. It allows for the creation of graded materials or functional gradients, where the composition and properties gradually change across the interface. This can be beneficial for specific applications that require tailored material properties. However, the abrupt change in composition and properties at the interface may lead to the weakening of the joint, thereby reducing the mechanical strength or introducing susceptibility to cracking or delamination. Additionally, the presence of intermetallic compounds within the unmixed zone can affect the corrosion resistance or ductility. By carefully controlling the energy input and the interaction between the different metals, it is possible to promote diffusion and reduce the formation of distinct interfaces. Additionally, post-processing techniques such as heat treatment or surface treatment processes can be applied to further refine the microstructure and properties of the printed part [28,29].

For laser cladding of one metal onto another dissimilar metal by the powder bed fusion (PBF), researchers have analysed the microstructure, grain morphology, composition distribution and interlayer iso-surface morphology through laser scanning microscope, electron backscatter diffraction (EBSD) and energy dispersive spectrometry [30]. The research objects include Fe-based alloys (SS316L/Cu [31]), Ti-based alloys (Ti6Al4V/invar [32], Ti-6Al-4V/304L [33], et.), Cu-based alloys (Cu10Sn/PA11 [34], Cu/Al [35], Cu/Zr [36]) and other related alloys (Invar 36/V). These studies are helpful for researchers to analyse the macroscopic mechanical properties of dissimilar metals from a microscopic perspective. It is found that the intermetallics exhibiting thermal mismatch along the dissimilar alloy transition region (the interface zone) can cause cracks [37]. Intermetallics are a specific class of metallic compounds that exhibit ordered atomic arrangements and specific stoichiometry. They are different from traditional metal alloys, which typically have disordered atomic arrangements. LPBF enables the production of intermetallics by selectively melting and solidifying metal powders, including those containing intermetallic phases. Researchers have tried to construct a complex spatial composition distribution of alloys to improve the bonding strength of the dissimilar alloy joining regions [38]. However, as there are uncertainties in the deposition mechanism, the range of applicability of conventional LPBF is not as wide as directed energy deposition for multimetal processing.

**Figure 3:**
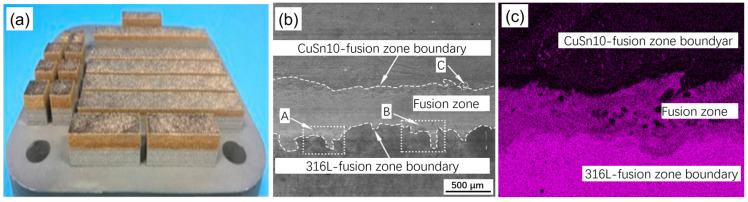
(**a**) Sample of powder bed fusion 316L/CuSn10; (**b**) scanning electron microscopy images indicating interface microstructure of laser powder bed fusion 316L/CuSn10; and (**c**) energy dispersive X-ray spectroscopy obtained a schematic diagram of component distribution near the interface [4].

Good metallurgical bonding characteristics were found in SS316L and C18400 Cu alloys. This is evident by the diffusion of both the materials as seen at the fused junction. The scattering of elements occurs due to the convective forces in the melt pool caused due to supercooling. This means that the rate of heat removal is higher than the heat of fusion releasing rate during the rapid cooling in LPBF [4]. Trends shown between SS316L and Cu10Sn are similar to the SS316L and C18400 Cu alloys. However, for SS316L, microscopic cracks were observed close to the boundary of the interface. However, for Cu10Sn, no microscopic cracks were observed near the boundary of the interface. Optimized variables for SS316L and Cu10Sn were used during the process of LPBF. Microscopic cracks occurred due to the difference in the coefficient of thermal expansion between steel and bronze, thus resulting in the formation of splits between them. Dendritic cracks also occur because of the high thermal conductivity of Cu10Sn (Figure 3). The high concentration of heat in the interface leads to a higher thermal stress and can cause cracks to grow in a perpendicular direction with respect to the boundary of the interface. In the SS316L-Cu10Sn multimetal parts, microhardness progressively reduces from SS316L to Cu10Sn. Similar observations are seen for SS316L and C18400 material combination. By utilizing optimized variables for interface formation, we can obtain good interfacial bonding with less cracks. In bending and shear directions, it is confirmed that multimetal specimens can resist a certain extent of torsional stress.

Multi-material processing in SLM using AlSi10Mg and UNS C18400 copper alloys was carried out [39]. Al_2_Cu intermetallic compound was formed at the Al/Cu bond interface after the SLM process. The tensile strength of Al/Cu SLM parts was evaluated to be 176 ± 31 MPa, and flexural strength under a 3-point bending test was evaluated to be around 200 MPa for Cu at root and 500 MPa for Al at root. Further analysis suggested that the formation of intermetallic compounds translated the fracture mechanism at the interface from ductile to brittle cleavage. The movement of elements via diffusion causes well-defined copper-rich and aluminum-rich parts with an intermixed part at the transition zone that can be identified by utilizing focused ion beam (FIB) imaging. The initial distribution of these elements may be uniform or varied across different regions of the part. As the laser or electron beam selectively melts the metal powder, localized heating and rapid solidification takes place. During the melting process, the high temperatures cause the atoms of the metal powders to become mobile. The melted region experiences a thermal gradient, with higher temperatures at the centre of the melt pool and lower temperatures at the periphery. This thermal gradient drives the diffusion of atoms from the high-temperature region to the low-temperature region. As the metal powder cools and solidifies, the atoms migrate from the high-concentration regions to the low-concentration regions through atomic diffusion. In the case of copper and aluminium, copper atoms tend to diffuse towards the aluminium-rich regions, and aluminium atoms tend to diffuse towards the copper-rich regions. The diffusion process continues during the solidification of subsequent layers as the build progresses [40,41]. Over time, the movement of atoms due to diffusion results in the formation of well-defined copper-rich and aluminium-rich regions within the printed part. The intermixed part at the transition zone represents the area where the diffusion of atoms from both materials is still occurring, thus leading to a gradual blending of the two elements. To analyse and visualize these compositional variations, techniques such as FIB imaging can be employed. FIB imaging allows for cross-sectional analysis of the printed part, thus revealing the distinct copper-rich and aluminium-rich regions as well as the transitional intermixed zone. The movement of elements through diffusion in multimetal PBF is influenced by various factors, including the temperature gradients, composition of the metal powders, and the process parameters employed. Optimizing these factors can help control the diffusion behaviour and achieve desired compositional variations and gradients within the printed part [42].

**Figure 4:**
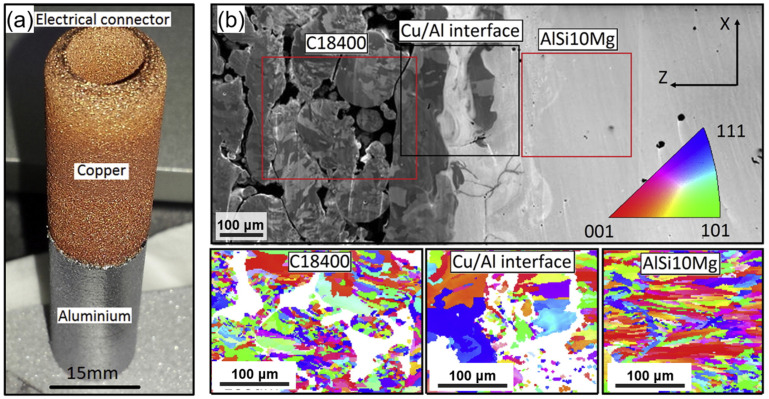
(**a**) Sample of powder bed fusion Cu/Al; (**b**) interface microstructure between Cu and Al obtained by scanning electron microscopy [35].

Just like SS316L/Cu10Sn, AlSi10Mg/C18400 copper alloy was also identified with cracks in segments of the interface which is also caused by different thermal coefficients [35]. When Al_2_Cu intermetallic compounds form, crack formation is aggravated, which was observed by using X-ray Diffraction. Intermetallic compounds are formed in the transition zone leading to anomalous readings for microhardness. This is caused by the precipitation of compounds that are tougher even though they are more brittle; see Figure 4.

### 3.2. Numerical Simulation

**Figure 5:**
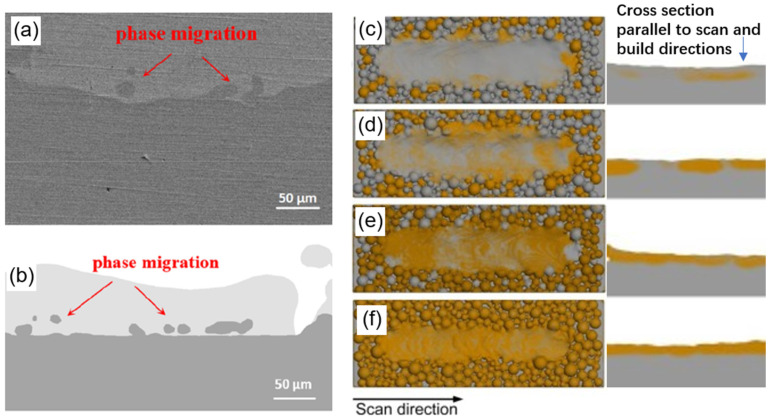
Interface shape between 316L and Cu10Sn produced by powder bed fusion: (**a**) experimental results, (**b**) simulation results [4]. Numerical simulation component distribution in multimetal powder bed fusion with different mixture powder (IN718/Cu10Sn): (**c**) 75% IN718, (**d**) 50% IN718, (**e**) 25% IN718 and (**f**) 0% IN718 [56].

**Figure 6:**
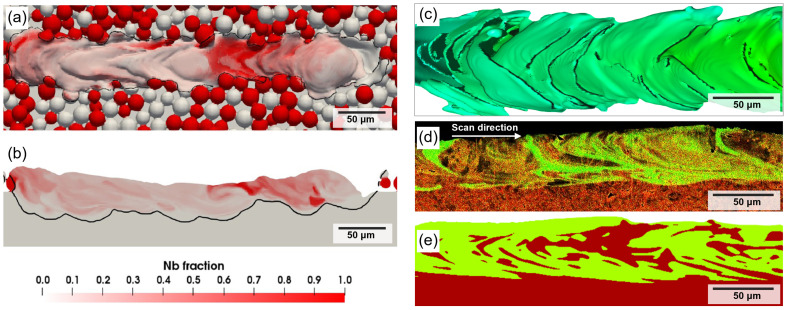
Numerical simulation distribution of Nb during powder bed fusion of Ti and Nb powders: (**a**) top view and (**b**) longitudinal cross-sectional view [57]. Interface shape and component distribution during printing IN718 powder on 316L substrate by powder bed fusion, (**c**) interface shape, (**d**) longitudinal cross-sectional view experimental results and (**e**) longitudinal cross-sectional view simulation results [58].

Reliable numerical models for metal additive manufacturing help to optimize the quality of the fabricated parts [43,44]. At present, for AM simulation, a computational fluid dynamics method (CFD) can consider most of the physical issues such as melting and solidification, evaporation and condensation, and multiphase flow in the additive manufacturing process [45]. Through fine calculation of surface tension [46], marangoni force [47], recoil pressure [48,49], magnetic field force [50] and damping force of the mushy zone [51], a detailed description on the complex flow of the metal liquid such as the keyholes, hole defects and splashing can be obtained [52]. Compared with other simulation methods, it has various advantages [53,54]. Recently, Gu and Sun et al. [55,56] used experimental and simulation methods to laser-clad Cu10Sn powder onto 316L substrate and IN718 substrate and found that the migration and diffusion of alloy components near the interface are related to the melt pool flow. In their simulation, the Cu10Sn powder on the top has a low laser absorption (about 0.03, less laser energy absorption makes the powder difficult to melt), is difficult to melt, so there is no keyhole, or the keyhole does not penetrate the interface of the two alloys, resulting in the substrate (SS316L or IN718) being melted by the thermal conduction of the powder (Cu10Sn). These factors cause the recoil pressure not to penetrate through the dissimilar alloy interface, and the Marangoni force has almost no effect on the alloy interface. As a result, the dissimilar alloy interface has a small and irregular wavy shape (Figure 5). Tang et al. [57] proposed an SLM computational framework for simulating multiple materials, accounting for the physical details of multi-physics and multimetals such as composition diffusion. Through simulation and experiments, Yao et al. [58,59] found that when IN718 powder is laser cladded on 316L substrate, the interface between IN718 and SS316 appears like a “fish scale” structure and produces curved grains. However, Yao et al. did not explain the dissimilar materials’ melt pool flow from the conduction mode to the keyhole mode (Figure 6). It is worth noting that the study by Yusuf et al. [60] showed that there are curved grains in the transition region between the IN718 powder and SS316 substrate. Since the alloy composition distribution of the fish scale shape is destroyed during the multilayer printing process, curved grains with a large curvature close to 180° are rarely observed.

## 4. Challenges in Multimetal Additive Manufacturing

### 4.1. Material Compatibility

**Figure 7:**
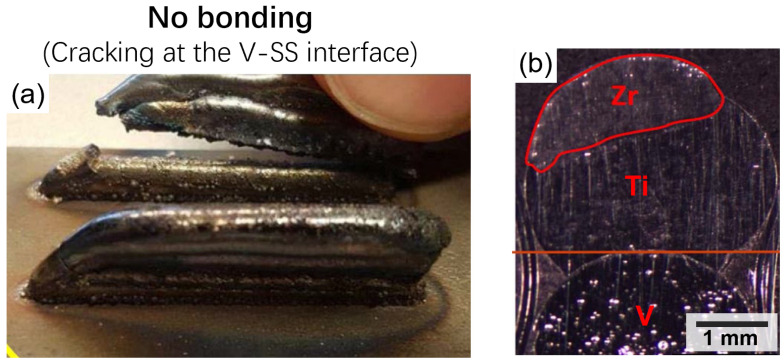
(**a**) Deposition of Zr on the A410-L stainless steel substrate by using the titanium and vanadium inter-layers. (**b**) Optical cross-section of produced failed dissimilar wall [36].

Material compatibility is a critical factor in multimetal additive manufacturing. Materials have varying physical and chemical properties. If they are not carefully selected and designed, the resulting object may not function as intended [16]. The materials must be compatible in terms of melting points, adhesion, and reactivity, among other factors. If the materials are not compatible, it can lead to delamination, cracks, or weak bonding between the layers, thus resulting in failed prints (Figure 7) [61]. The selection of materials must be based on several factors, including the intended application, the properties required for the object, and the compatibility with the printing process. The materials must also be tested to ensure that they are compatible with each other [62].

### 4.2. Porosity

Porosity is one of the most significant challenges during multimetal additive manufacturing (MMAM). Porosity occurs when there is insufficient bonding between layers or materials, leading to the formation of voids or gaps. Porosity can result from a range of factors, including material properties, printing parameters, and design of the part [63]. Materials with low viscosity and high surface tension are more prone to porosity, as they tend to bead up and form gaps between layers. Poor adhesion between layers and air entrapment within the material can also lead to porosity [64]. Porosity can affect the mechanical properties and performance of the final product, leading to reduced strength, stiffness, and durability. Porous parts are more prone to cracking, deformation, and failure, and can have a shorter lifespan than non-porous parts. Porosity can also affect the aesthetics of the part, leading to rough or uneven surfaces that are unsuitable for certain applications [65].

**Figure 8:**
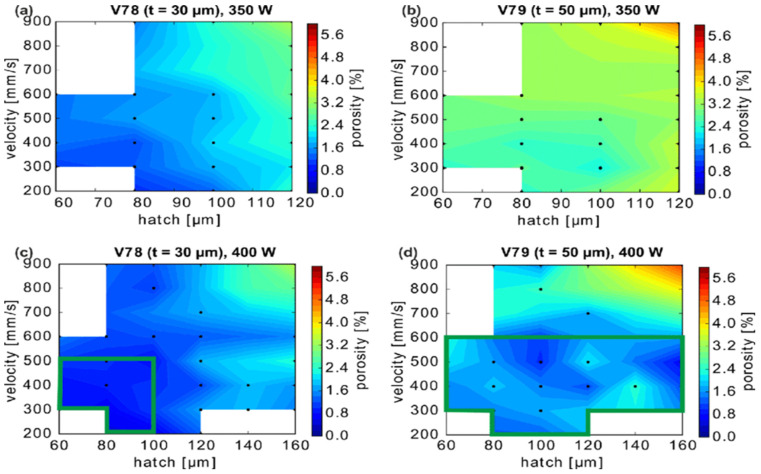
CuCrZr porosity dependence on layer height (t). The white region represents the parameter combination not measured and the black dots represent the measured process parameters. The green rectangles depict minimum porosity percentage [69].

Several solutions have been proposed to address the challenges of porosity during MMAM [66]. The use of a higher printing temperature can help to improve adhesion between layers, while the use of a lower printing speed can help to reduce air entrapment and improve layer bonding [67]. The use of a lower layer height can also help to reduce porosity (Figure 8). It increases the surface area of each layer, thereby promoting better bonding. From Figure 8, it is evident that the porosity percentage is lesser at a layer height (t) of 30 µm compared to a layer height of 50 µm. As the laser or electron beam melts the powder, the finer particles have a higher chance of fusing together, thereby creating a denser and more solid layer. By promoting better bonding between the particles, a lower layer height reduces the likelihood of the formation of voids and minimizes the porosity in the final printed part. This can lead to denser and more structurally sound components, while also enabling higher resolution and finer detail in the printed part. However, careful consideration of process parameters is necessary to manage potential challenges associated with thinner layers [68].

Another potential solution is the use of support materials. Support materials can help to reduce porosity by providing additional support during the printing process. Support materials with a high thermal conductivity can facilitate efficient heat dissipation during the laser or electron-beam scanning process. By absorbing and conducting heat away from the melt pool, they can help maintain a stable temperature gradient and reduce the formation of porosity associated with excessive heat accumulation. Support materials can contribute to the compaction of the metal powder layers. As the layers are deposited and compacted, the support material aids in minimizing voids and gaps between the powder particles [25]. This promotes better powder fusion and reduces the likelihood of porosity formation during the melting and solidification stages. It provides mechanical support to the partially melted or overhanging sections of the printed part. By preventing deformation or sagging during the melting process, support materials help maintain the integrity of the part and minimize the formation of porosity caused by instability or structural collapse. They can also assist in managing the thermal stresses that arise during the cooling and solidification of the printed part. By absorbing or counteracting the thermal stresses, support materials help prevent warping, cracking, and the formation of porosity due to stress-induced defects. They can influence the heat transfer dynamics during the additive manufacturing process. They can act as a thermal barrier, controlling the rate of heat transfer to adjacent regions of the part. This controlled heat transfer can mitigate the formation of thermal gradients, which can lead to porosity, and promote more uniform solidification [70]. For instance, a sacrificial support material can be printed alongside the main material, which can be removed after printing.

### 4.3. Cracks

**Figure 9:**
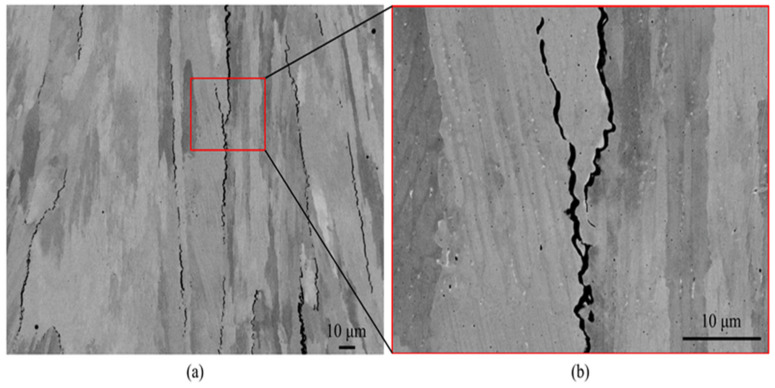
(**a**) Crack morphology and (**b**) enlarged crack morphology developed along grain boundaries of stainless steel 316L fabricated via laser powder bed fusion [73].

Cracks are a significant challenge during MMAM and can occur due to a range of factors, including material properties, printing parameters, and the design of the part. The use of multiple metals in a single process can exacerbate the formation of cracks, as each material has its own properties and behaviour. Metals with different thermal expansion coefficients can cause differential contraction and expansion during the cooling process, leading to cracks in the printed part [71]. Poor adhesion between layers or materials can also lead to the formation of cracks (Figure 9). It can also be caused due to improper cooling or support structures. Cracks can affect the mechanical properties and performance of the final product, leading to reduced strength, stiffness, and durability [72]. Cracked parts are more prone to failure and can have a shorter lifespan than non-cracked parts. Cracks can also affect the aesthetics of the part, leading to rough or uneven surfaces that are unsuitable for certain applications.

Several solutions have been proposed to address the challenges of cracks during MMAM. The printing parameters, such as temperature, speed, and layer height, can be adjusted to reduce the likelihood of cracking [36]. For instance, the use of a lower printing speed can help to reduce thermal stress and improve bonding between layers, while the use of a higher printing temperature can help to reduce the viscosity of the material and improve layer bonding. Another potential solution is the use of support structures. Support structures can help to reduce the formation of cracks by providing additional support during the printing process [74]. Support structures can help to prevent deformation and sagging during the printing process, leading to better bonding between layers and reducing the formation of cracks. Support structures can be printed using the same or different material as the main part and can be removed after printing. The use of post-processing techniques can also help to reduce the formation of cracks. Post-processing techniques such as annealing or coating can help to improve the bonding between layers and reduce the likelihood of cracks. Annealing involves applying heat to the printed part, which can help to reduce thermal stress and improve the bonding between layers [75]. Coating involves applying a layer of material on top of the printed part, which can help to improve the aesthetics and reduce the formation of cracks.

### 4.4. Loss of Alloying Elements

**Figure 10:**
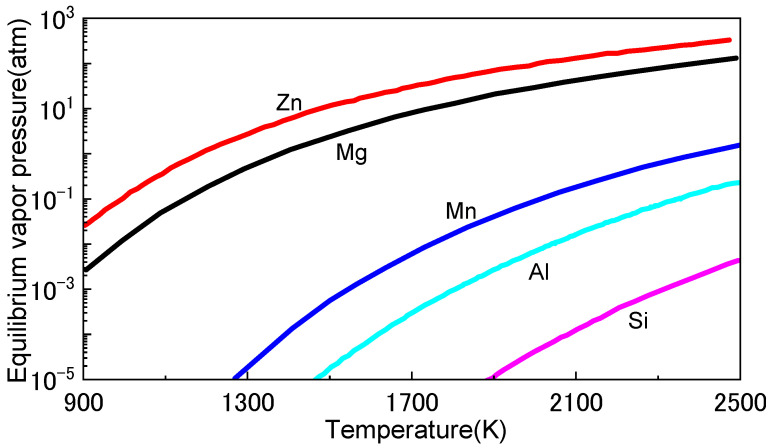
Equilibrium vapour pressure of metals at different temperatures [78].

The loss of alloying elements is a critical challenge during MMAM, particularly in the printing of metal alloys. The printing process can lead to the loss of alloying elements through evaporation or oxidation, which can affect the mechanical properties and performance of the final product. Alloying elements are added to metals to improve their mechanical properties, such as strength, stiffness, and corrosion resistance. The loss of these elements can lead to reduced strength, stiffness, and corrosion resistance, and can compromise the integrity and reliability of the final product. The loss of alloying elements can occur due to several factors, including printing parameters, material properties, and design of the part. For instance, high temperatures and long printing times can lead to the loss of alloying elements through evaporation or oxidation [76]. Materials with high vapor pressures (Figure 10) are particularly susceptible to the loss of alloying elements. The design of the part can also affect the loss of alloying elements, as certain geometries can trap gases or promote oxidation, thus leading to the loss of alloying elements [77].

Several solutions have been proposed to address the challenges of the loss of alloying elements during MMAM. One of the most effective solutions is the optimization of printing parameters [79]. For instance, the use of a lower printing temperature can help to reduce the evaporation or oxidation of alloying elements, while the use of a controlled atmosphere, such as a protective gas, can help to reduce the oxidation of alloying elements. Another potential solution is the use of pre-alloyed powders or wires. Pre-alloyed powders or wires contain a homogeneous mixture of alloying elements and the base metal, which can reduce the likelihood of the loss of alloying elements during the printing process. Pre-alloyed powders or wires can be used in PBF techniques and can help to improve the mechanical properties and performance of the final product [80]. The use of post-processing techniques can also help to reduce the loss of alloying elements. Post-processing techniques such as annealing or heat treatment can help to reduce the loss of alloying elements by promoting diffusion and re-alloying of the printed part. Annealing involves applying heat to the printed part, which can help to promote diffusion. Heat treatment involves exposing the printed part to a specific temperature and atmosphere, which can help to promote diffusion and re-alloying of the printed part.

### 4.5. Oxide Inclusions

Oxide inclusions are also a critical challenge during MMAM, particularly in the printing of metal alloys. The printing process can lead to the formation of oxide inclusions due to several factors, including the high temperatures involved and the use of reactive materials. Oxide inclusions can affect the mechanical properties, such as strength and fatigue resistance, and can also lead to cracks or voids in the final product [81]. Oxide inclusions can also affect the surface finish and appearance of the final product, making it unsuitable for certain applications, such as aerospace industries. The presence of oxide inclusions can also lead to porosity, which can affect the integrity and reliability of the final product. The formation of oxide inclusions can be difficult to predict and control, making it a significant challenge in the field of MMAM [82].

Several solutions have been proposed to address the challenges of oxide inclusions during MMAM. One of the most effective solutions is the use of inert or reducing atmospheres during the printing process [83]. Inert atmospheres, such as argon or nitrogen, can help to prevent the formation of oxide inclusions by displacing the oxygen in the printing chamber. Reducing atmospheres, such as hydrogen or carbon monoxide, can also help to reduce the formation of oxide inclusions by reacting with the oxygen to form water or carbon dioxide. Post-processing techniques can also help to reduce the formation of oxide inclusions. Techniques such as hot isostatic pressing (HIP) or hot forging can help to promote diffusion and bonding between the particles, which can reduce the formation of oxide inclusions. HIP involves subjecting the printed part to high temperatures and pressures to compress and bond the particles, while hot forging involves pressing and shaping the printed part at high temperatures to promote bonding [84].

### 4.6. Interfacial Phases and Unmelted Particles

**Figure 11:**
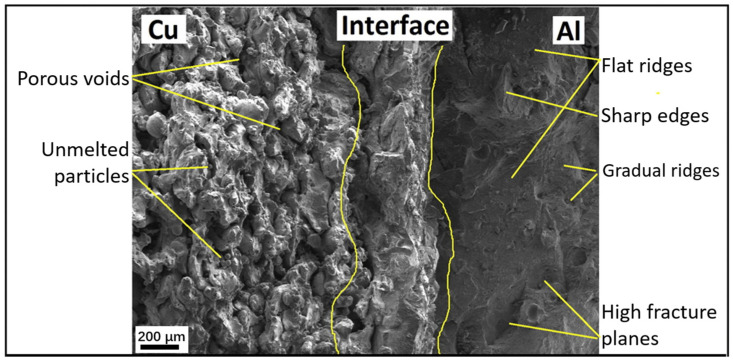
Fracture morphology showing the unmelted particles near the Cu-Al interface [17].

Interfacial phases and unmelted particles can form at the interface between two materials during the printing process (Figure 11). These phases can lead to the formation of weak bonds between the materials, leading to poor mechanical properties and reduced reliability. Interfacial phases and unmelted particles can also lead to voids or cracks, which can further compromise the final product’s integrity [62]. The formation of interfacial phases and unmelted particles can be due to several factors, including the materials’ properties and the printing parameters. For example, the presence of incompatible materials or the use of high laser power can lead to the formation of interfacial phases and unmelted particles. The printing process’s speed and temperature can also affect the formation of these phases [85].

Several solutions have been proposed to address the challenges of interfacial phases and unmelted particles during MMAM. One of the most effective solutions is the use of interlayers or transition layers between the materials to promote bonding. Interlayers can help to minimize the formation of interfacial phases and unmelted particles by providing a gradual transition between the two materials. The use of multiple lasers can also help to reduce the formation of interfacial phases and unmelted particles. Multiple lasers can enable the printing of multiple materials simultaneously, reducing the chances of interfacial phases forming between the materials. Multiple lasers can also help to reduce the printing time, improving the productivity of the printing process [86]. Post-processing techniques can also help to reduce the formation of interfacial phases and unmelted particles.

### 4.7. Post-Processing

#### 4.7.1. Thermal and Mechanical Mismatch

Post-processing of multimetal parts is challenging due to the thermal and mechanical mismatch of the materials used. The materials used in MMAM have different melting points, coefficients of thermal expansion, and elastic moduli. Therefore, heat treatment, finishing, and polishing processes can affect the materials differently, leading to distortion, warping, or cracking. This challenge can be addressed by developing post-processing techniques that can treat different metals uniformly, such as laser shock peening, annealing, or hot isostatic pressing [87].

#### 4.7.2. Surface Finishing

Surface finishing is an essential step in the post-processing of MMAM parts as it improves their aesthetic appeal and functional performance. However, the surface finishing of multimetal parts can be challenging due to the different material properties and geometries. The use of traditional polishing techniques can lead to uneven surfaces, roughness, or damage to the weaker materials. This challenge can be addressed by developing new surface finishing techniques such as electrochemical polishing, abrasive flow machining, or laser-assisted polishing [88].

#### 4.7.3. Residual Stresses

Residual stresses can develop in multimetal parts during the printing process and post-processing due to the different thermal and mechanical properties of the materials. These stresses can lead to warping, distortion, or cracking of the parts, reducing their mechanical properties and performance. This challenge can be addressed by developing post-processing techniques that can relieve the residual stresses uniformly, such as shot peening, thermal stress relief, or vibratory stress relief [89]. Shot peening is a post-processing technique that involves bombarding the surface of the printed part with small spherical media, typically metal or ceramic particles. The impact of the particles induces plastic deformation and introduces compressive stresses on the surface, thus helping to counterbalance the tensile residual stresses within the part. This process improves the fatigue life and dimensional stability of the component. It is effective in reducing residual stresses in localized regions of the part, such as notches or critical stress-concentration areas [90]. Thermal stress relief is a post-processing technique that utilizes controlled heating and cooling to relieve residual stresses [25]. The part is heated to a temperature below its melting point but above the annealing temperature of the material. This allows for the redistribution of stresses through plastic deformation and creep. The part is then slowly cooled to room temperature, thus allowing the stresses to relax. Thermal stress relief is effective in reducing residual stresses across the entire part, thereby promoting dimensional stability and reducing the risk of warping or distortion. However, this method may not be suitable for components with complex geometries or sensitive features, as it can cause shape changes or material degradation. Vibratory stress relief (VSR) is a technique that involves subjecting the printed part to controlled mechanical vibrations. The vibrations induce micro-plastic deformation within the part, thus leading to stress relaxation and redistribution. It can be performed at room temperature and is considered a non-destructive method for stress relief. It is particularly useful for large or complex parts that may be difficult to treat using other techniques. It can also help to minimize warping, distortion, and residual stresses while preserving the shape and dimensional accuracy of the printed component.

#### 4.7.4. Corrosion and Wear Resistance

Corrosion and wear resistance are essential properties of metal components in various applications such as aerospace, automotive, and biomedical. However, the different material properties and geometries of multimetal parts can make it challenging to develop post-processing techniques that can improve their corrosion and wear resistance uniformly. This challenge can be addressed by developing new surface modification techniques such as chemical passivation, plasma nitriding, or electroless plating [91].

A summary of all these challenges along with their solutions is given in Table 1.

## 5. Potential Research for Multimetal Additive Manufacturing

Material compatibility: Material compatibility is a major challenge in multimetal PBF. The use of dissimilar metals in this technique can result in interfacial phase formation, which can affect the properties of the final product. Future research could focus on developing new materials that are compatible with each other, ensuring a reduction in interfacial interaction between dissimilar materials, or on developing coatings that can prevent interfacial phase formation [71].

Post-processing: Post-processing is an important aspect of multimetal PBF. The final product often requires finishing operations such as polishing, heat treatment, and coating. Future research could focus on developing new post-processing techniques that are specific to multimetal PBF. For example, new polishing techniques could be developed that are suitable for multiple metals, or new heat-treatment methods could be developed that can reduce interfacial phase formation [1,92].

In situ monitoring: In situ monitoring is an important aspect of multimetal PBF. In situ monitoring can provide real-time feedback on the process, thereby enabling the operator to adjust the process parameters in real-time. New sensors could be developed that can monitor the composition of the final product, or new imaging techniques could be developed that can provide real-time feedback on the process [93].

Multi-scale modelling: Multi-scale modelling is an important aspect of multimetal PBF. Multi-scale modelling can provide insights into the microstructure and properties of the final product, thus enabling the design of new materials and processes [57].

## 6. Future Prospects of Multimetal Additive Manufacturing

Development of new materials. Multimetal additive manufacturing has the potential to enable the development of new materials with improved properties.

- Metal composites: PBF allows for the integration of different metal powders, thus enabling the fabrication of metal composites. These composites can combine metals with contrasting properties, such as high strength and high conductivity, to create materials with enhanced performances. For example, combinations of copper and steel, or aluminium and titanium, can be printed to achieve a balance between strength, conductivity, and lightweight properties.
- Functionally graded materials (FGMs): PBF can be used to fabricate FGMs by varying the composition of the metal powders layer by layer. This opens up possibilities for creating materials with tailored properties, such as thermal gradients, hardness variations, or corrosion resistance. For instance, a transition from stainless steel to nickel alloy can be achieved, thereby providing resistance to both high temperatures and corrosive environments.
- Multi-alloy structures: PBF allows for the printing of multiple alloys within the same part. This capability can be leveraged to create complex structures with localized variations in material properties. For example, different sections can be printed within a single component using alloys with varying hardness, wear resistance, or thermal conductivity. This enables the design of components with optimized performance in specific regions.
- Intermetallic combinations: PBF enables the fabrication of intermetallic combinations, wherein dissimilar metals form compounds with specific crystal structures and properties. By carefully controlling the composition and processing parameters, intermetallic combinations such as aluminium–nickel, copper–zinc, or iron–nickel can be printed. These intermetallic materials can exhibit unique mechanical, thermal, and electrical properties that are distinct from those of the constituent metals.
- Bi-metallic joints: PBF can be used to create strong and reliable joints between dissimilar metals. By selectively melting and bonding different metals, PBF allows for the creation of bi-metallic joints with high bonding strength and integrity. Such joints find applications in industries such as aerospace and automotive, where dissimilar metals need to be securely joined for specific functional requirements.

This technology allows for the combination of metals and alloys in various proportions, leading to the fabrication of materials with tailored properties. The development of new materials is expected to expand the range of applications for additive manufacturing [94].

Increased adoption in aerospace industry. The aerospace industry has been an early adopter of additive manufacturing, and MMAM is expected to gain significant traction in this sector. The ability to fabricate complex geometries with multiple metals and alloys in a single build opens new possibilities for the manufacturing of aircraft components. For instance, MMAM can be used to fabricate engine parts with improved mechanical properties, which are critical to the performance and safety of the aircraft.

Medical applications. Multimetal additive manufacturing is expected to have a significant impact on the medical industry. The technology has the potential to fabricate custom implants with tailored mechanical properties and geometries. For instance, MMAM can be used to fabricate orthopaedic implants with a combination of titanium and cobalt-chromium, leading to improved biocompatibility and mechanical properties. The technology can also be used to fabricate medical devices, such as surgical tools and prosthetics, with intricate details and complex geometries [95].

Mass production of consumer goods. Multimetal additive manufacturing has the potential to revolutionize the mass production of consumer goods. The technology allows for the rapid production of complex geometries with multiple metals and alloys in a single build, thus leading to cost savings and reduced lead times. The development of new materials and the ability to fabricate complex geometries are expected to expand the range of consumer goods that can be manufactured using additive manufacturing [96].

Integration with other technologies. Multimetal additive manufacturing is expected to be integrated with other technologies, such as artificial intelligence and robotics. The integration of these technologies is expected to improve the efficiency and accuracy of the manufacturing process.

## 7. Conclusions

Multimetal additive manufacturing, which involves printing components with multiple metals or alloys, is a promising technology. It allows for the creation of complex, multi-functional parts with tailored material properties. Powder bed fusion (PBF) technology is a widely used method for metal 3D printing. L-APBF is a variation of LPBF that employs a larger laser beam spot size, thus covering a larger area during each scan. Microstructural analysis techniques play a crucial role in characterizing additively manufactured components. There are also several challenges that need to be addressed to realize the full potential of MMAM. Material compatibility is a significant concern, as different metals may have different melting points and thermal properties, thus making it challenging to achieve a homogeneous blend of materials during printing. Irregularly shaped molten metal droplets may also form during the printing process. This can lead to defects and poor part quality. We must overcome these challenges to enhance the capabilities of multimetal additive manufacturing. Improving material compatibility through alloy development and optimization of process parameters is essential. Balling may be addressed by optimizing laser or electron beam parameters, modifying powder characteristics, or employing process monitoring and feedback control systems. The field of multimetal additive manufacturing is rapidly evolving and holds significant potential for innovation across various industries. As the technology continues to advance, it will open new possibilities for creating complex, customized, and high-performance components. We must continuously develop multimetal additive manufacturing processes to expand its potential applications.

## Figures and Tables

**Table 1:** Summary of the challenges in multimetal additive manufacturing via powder bed fusion and their solutions.

Challenge	Solution
Material Compatibility	Selection of compatible materials, optimization of printing parameters, post-processing techniques such as diffusion bonding or brazing
Porosity	High printing temperatures, lower printing speeds, lower layer height, use of support materials
Cracks	Optimization of printing parameters, use of support structures, post-processing techniques such as annealing and coating
Loss of Alloying Elements	Optimization of printing parameters, use of pre-alloyed powders or wires, post-processing techniques such as annealing or heat treatment
Oxide Inclusions	Use of inert or reducing atmospheres, post-processing techniques such as hot isostatic pressing (HIP) or hot forging
Interfacial Phases and Unmelted Particles	Use of interlayers or transition layers, using multiple lasers, post-processing techniques such as hot isostatic pressing (HIP) or hot forging
Thermal and Mechanical Mismatch	Laser shock peening, annealing, hot isostatic pressing
Surface Finishing	Electrochemical polishing, abrasive flow machining, laser-assisted polishing
Residual Stresses	Shot peening, thermal stress relief, vibratory stress relief
Corrosion and Wear Resistance	Chemical passivation, plasma nitriding, electroless plating

## Data Availability

Not applicable.

## Abbreviations

PBF	Powder bed fusion
LPBF	Laser powder bed fusion
EB-PBF	Electron beam—powder bed fusion
L-APBF	Large area pulsed laser powder bed fusion
SLM	Selective laser melting
GNS	Gradient nanostructured
FGM	Functional gradient materials
DMLS	Direct metal laser sintering
SLS	Selective laser sintering
EBM	Electron beam melting
FIB	Focussed ion beam
MMAM	Multimetal additive manufacturing
SEM	Scanning electron microscopy
HIP	Hot isostatic pressing
